# Angle-specific analysis of isokinetic quadriceps and hamstring torques and ratios in patients after ACL-reconstruction

**DOI:** 10.1186/s13102-018-0112-6

**Published:** 2018-12-06

**Authors:** Christian Baumgart, Wouter Welling, Matthias W. Hoppe, Jürgen Freiwald, Alli Gokeler

**Affiliations:** 1 Department of Movement and Training Science, University of Wuppertal, Fuhlrottstraße 10, 42119 Wuppertal, Germany; 2 Center for Human Movement Science, University of Groningen, University Medical Center Groningen, Antonius Deusinglaan 1, 9713 AV Groningen, The Netherlands; 3 Medisch Centrum Zuid, Sportlaan 2-1, 9728 PH Groningen, The Netherlands; 4 Department of Orthopedic, Trauma and Hand Surgery, Klinikum Osnabrück GmbH, Osnabrück, Am Finkenhügel 1, 49076 Osnabrück, Germany; 5 Exercise Science and Neuroscience, Department of Exercise & Health, University of Paderborn, Warburger Str. 100, 33098 Paderborn, Germany; 6 Luxembourg Institute of Research in Orthopedics, Sports Medicine and Science, Luxembourg, Luxembourg

**Keywords:** Anterior cruciate ligament, Rehabilitation, Team sports, Return-to-sport (RTS), Isokinetic strength

## Abstract

**Background:**

Strength deficits, muscle imbalances, and quadriceps inhibition are common after the surgical reconstruction of the anterior cruciate ligament (ACL), even after the patient’s returned-to-sport. Typically, asymmetries between the operated and non-operated leg as well as the hamstring/quadriceps (HQ) ratio are calculated using maximum isokinetic torque values. Moreover, the knee flexion angles, which correspond to the measured torque values, were not considered. Therefore, the aim of the study was to evaluate the usage of an angle-specific approach for the analysis of isokinetic data in patients after an ACL-reconstruction.

**Methods:**

A cross-sectional laboratory study design was used to investigate the influence of leg (operated vs. non-operated) and two velocities on angle-specific isokinetic data. Concentric quadriceps and hamstring torques and ratios of 38 patients were assessed 6.6 months after ACL-reconstruction with a hamstring tendon graft. At a velocity of 60°/s and 180°/s, angle-specific torques and HQ-ratios were analyzed with conventional discrete parameters and a Statistical Parametric Mapping procedure, which evaluates continuous data. The relationship between angle-specific and conventional HQ-ratios was evaluated with Pearson correlation coefficients.

**Results:**

Angle-specific torques and HQ-ratios were different between the operated and non-operated leg and between velocities. In the operated leg, the quadriceps deficit was higher at 60°/s in knee flexion angles > 50°. The HQ-ratios decreased with greater knee flexion at both velocities, but with a different magnitude. Around 30°, the HQ-ratios reached 1.0 and did not differ between the velocities, while leg differences were present from 40 to 60°. At the higher testing velocity, the maximum flexion torque occurred at greater knee flexion, whereas the maximum extension torque were present at a similar joint angle. The correlation coefficients between conventional and angle-specific HQ-ratios were low in knee angles < 35° and > 65° and varied according to leg and velocity.

**Conclusions:**

The angle specific approach is recommended for future ACL-research, as it reveals strength deficits and imbalances, which were not captured by conventional parameters. The results provide a rationale for more specific joint angle and/or velocity based training and may help for return-to-sport decisions.

## Key points

- The Statistical Parametric Mapping approach applied to isokinetic torque data reveals angle-specific strength deficits and imbalances, which were not captured by conventional parameters.
- Conventional data analysis potentially underestimate strength deficits in patients after an ACL-reconstruction, and may have led to questionable conclusions or practical applications in the past.
- The angle-specific analysis of isokinetic torque data provides a rationale for more specific joint angle and/or velocity based training and may also helpful for future return-to-sport decisions.

## Background

Strength deficits, muscle imbalances, and quadriceps inhibition are common after the surgical reconstruction of the anterior cruciate ligament (ACL), even after the patient’s returned-to-sport (RTS) [1, 2]. Moreover, strength deficits and muscle imbalances prior the RTS were associated with an increased risk of an ACL graft rupture [3, 4]. The assessment of quadriceps and hamstring strength in RTS settings is commonly done using isokinetic tests at velocities of 60°/s and 180°/s [5, 6]. Typically, asymmetries between the operated and non-operated leg as well as the hamstring/quadriceps (HQ) ratio are calculated using maximum torque values [5, 7, 8].

One criticism of conventional isokinetic analyses is the reduction of the continuous torque-time data to single values (e.g. maximum torque, HQ-ratio), potentially leading to a loss of information [9, 10]. For the statistical evaluation of continuous data, two statistical methods were recently established in human movement research, namely the *Functional Data Analysis (FDA)* and *Statistical Parametric Mapping (SPM)* [10]. Both methods have been applied in ACL-research to analyze movement strategies during cutting [11], stair descent [12], and unilateral and bilateral jumps [13, 14]. It was shown, that these approaches can reveal clinically meaningful information that cannot be captured with conventional calculated parameters, since they can account for separate movement phases. Therefore, the application of such methods may be also promising in isokinetic data analysis.

A further criticism of conventional isokinetic analyses is that they do not consider the knee flexion angles, which correspond to the measured torque values [15]. Therefore, maximum torque values provide only limited information about the muscle performance throughout the entire range of motion (ROM) [16]. To date, angle-specific torque values have been addressed by a few studies in healthy people [17–20] and patients [15, 16, 21, 22]. In ACL-deficient patients, greater quadriceps deficits were found in knee flexion angles between 0 and 40° at 60°/s [15, 16]. To the best of our knowledge, only two studies have investigated angle-specific analysis of isokinetic torque data in patients after an ACL-reconstruction [21, 22]. These studies found angle- and velocity-specific muscle imbalances between the operated leg of the patients and control legs [21, 22]. Moreover, compared to controls, the angle-specific HQ-ratios were overall increased; particularly, at full knee extension [21]. However, these two studies have used the same cohort of patients and included the acceleration and deceleration phases of the lever arm. This may had an impact on the results, because local extremes in torque at the start and end of each repetition were frequently discernible, which originate from inertial forces [23].

Despite the existing knowledge of isokinetic strength in patients after an ACL-reconstruction, the angle-specific analysis of isokinetic torque data could lead to a better understanding of the muscle adaptations during the rehabilitation process and could also be helpful for RTS decisions. Therefore, the aim of the study was to evaluate the usage of an angle-specific approach in patients after an ACL-reconstruction. It was hypothesized that isokinetic torques and HQ-ratios differ between the operated and non-operated leg, and further, that these differences vary according to the knee flexion angle and movement velocity.

## Methods

Thirty-eight team sport athletes participated in this study (18 females, 20 males), who had all an unilateral ACL-reconstruction using a hamstring tendon graft (Table 1). At the time of testing, all patients were between the 5.7 and 9.0 month post-surgery (Table 1). The rehabilitation protocol was standardized and has been described in detail earlier [6]. The study protocol was approved by the Medical Ethical Committee (ID 2012.362) of the University of Groningen. All patients were informed about the study procedures and have given their written consent to participate. Parental consent was given for patients under an age of 18 years (*n* = 3).

**Table 1:** Patient characteristics

Parameter	Mean ± sd (range)
Age [y]	25.1 ± 7.7 (16–47)
Body mass [kg]	72.3 ± 10.7 (53–95)
Post OP [month]	6.6 ± 0.7 (5.7–9.0)
IKDC score	81.4 ± 7.5 (52–94)
IKDC (z) score	−0.29 ± 0.44 (− 1.84–0.38)
Sport [n]	soccer 24; handball 7; basketball 3; volleyball 2; korfball 2

Before testing, all patients performed a 10 min warming-up procedure on a stationary bike. Isokinetic knee flexion and extension torques (Biodex System 3; Biodex Medical Systems, Inc., Shirley, NY) were tested for both legs at a velocity of 60°/s and 180°/s with five and ten maximal concentric repetitions, respectively. An average of three submaximal repetitions was performed to familiarize the patients with the testing protocol. The non-operated leg was tested first. There was a rest of 1 min between each trial. All tests were performed by the same researcher. A good to high test-retest reliability (ICC: 0.81 to 0.97) has been reported for isokinetic strength tests in patients after an ACL-reconstruction [24]. After testing, subjective evaluation of the knee function was assessed using the International Knee Documentation Committee Subjective Knee Form (IKDC), which was converted into a standard score (z) to permit a more valid comparison among patients, who differ regarding their age and sex [25].

Isokinetic data were measured with a sampling rate of 100 Hz, gravity-corrected according to the manufactures user guide, and normalized to body mass. Torque and velocity data were than filtered with a recursive second-order digital low-pass Butterworth filter using a cut-off frequency of 5 Hz. The repetitions were selected applying a torque threshold of 0.1 Nm/kg, and the first and final repetition were excluded from the analysis to avoid irregularities from the start and end of the test.

For the angle-specific analyses, all torque-, velocity-, and angle-time curves were cut at the start and end of each repetition, if the velocity was lower than 50°/s as well as 150°/s to neglect any inertia effects [23]. Therefore, the available ROM may differ for each repetition. Hence, a total of 1672 extension and flexion torque-angle curves were generated in steps of 1°, using a linear interpolation technique. After a visual inspection and consensual decision of two investigators, single repetitions (60°/s: flexion 8 rep., extension 7 rep.; 180°/s: flexion 1 rep., extension 2 rep.) were removed, because of non-typically shapes (e.g. due to a short ROM). Thereafter, the mean extension and flexion torque-angle curves were computed for each patient, leg, and velocity. These curves were additionally smoothed by a fifth-order Savitzky-Golay filter with a frame size of 21 and were than used to calculate the angle-specific HQ-ratios. A ROM of 19° to 81° was present in all patients, and therefore, entered in the statistical evaluation of the torque-angle and HQ-ratio curves.

Finally, the work, maximum extension and flexion torques and corresponding knee angle values were computed from the filtered data for each repetition, which was included in the angle-specific analyses. Therefore, the work was defined as the integral of the torque-angle curve.

Descriptive statistics were reported as mean values and standard deviations. The conventional calculated isokinetic parameters were checked for normal distribution with the Shapiro-Wilk test and were then analyzed with a two-factor (leg x velocity) repeated measure ANOVA. To evaluate the relationships between the conventional and angle-specific HQ-ratios Pearson correlation coefficients were calculated for each knee flexion angle and were plotted as correlation-angle curves. Moreover, two-factor (leg x velocity) repeated measure ANOVA’s of SPM were used to compare the angle-specific torque and HQ-ratio data. The scalar output statistic SPM{F} was calculated for the ROM of 19° to 81°, which allows the identification of significant different regions of the curves rather than only focusing on instant points of the signal [26]. The normality assumption of SPM was implicitly checked with the agreement between parametric and non-parametric results [9]. Statistical analyses were performed using R 3.1.2 [27] and the SPM analyses were implemented in Python using the open-source package spm1d (v. 0.4, www.spm1d.org). A level of *P* < 0.05 was set for statistical significance.

## Results

**Fig. 1:**
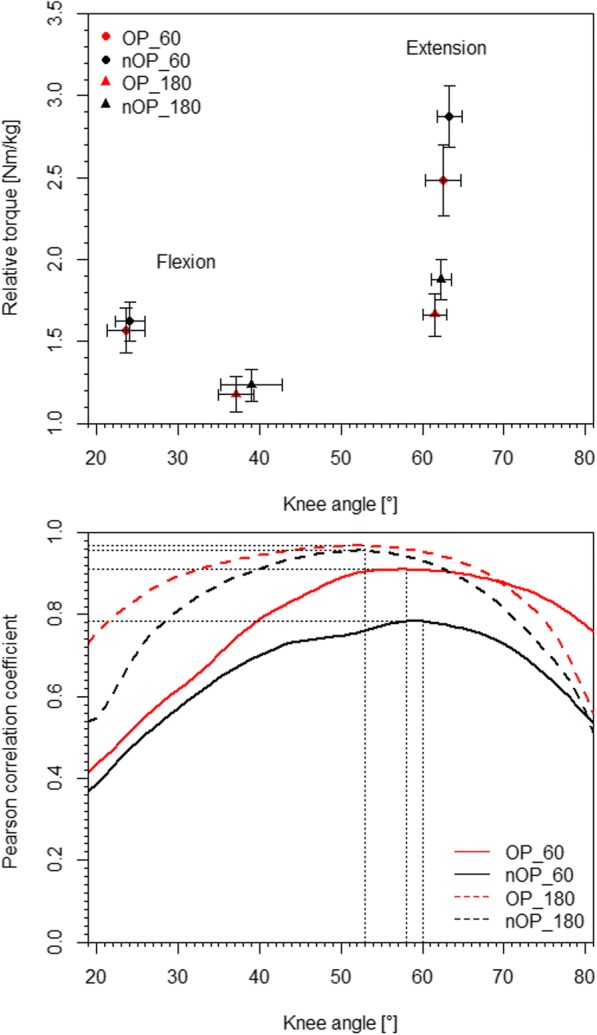
TOP: Maximum isokinetic torque and corresponding knee angle values (mean and 95% confidence intervals) separated for the operated (OP) and non-operated (nOP) leg as well as the 60°/s and 180°/s velocity. BOTTOM: Linear relationships between the conventional HQ-ratio and the angle-specific HQ-ratios separated for the operated (OP) and non-operated (nOP) leg as well as the 60°/s and 180°/s velocity. Note: dotted lines mark the locations of the maximum values

The mean IKDC and IKDC (z) score of the patients were 81.4 ± 7.5 and − 0.29 ± 0.44, respectively. Table 2 shows the results of the conventional isokinetic parameters. The location and amount of the maximum torque values separated for the operated and non-operated leg as well as the 60°/s and 180°/s velocity were separately presented in Fig. 1 (top). A main leg effect was found for all parameters with exception of the knee angle at the maximum torque during extension and flexion. In the operated leg, the torque and work values were significant reduced, while the HQ-ratios were higher. With exception of the angle at the maximum extension torque, main velocity effects were present in all parameters. Torque and work values were higher at 60°/s, while the knee angles at the maximum torque during flexion and the HQ-ratios were higher at 180°/s. A significant interaction effect was found for the maximum torque and work values during the extension, revealing that the leg differences at 60°/s were greater than those at 180°/s, and the differences between the two velocities were lower in the operated leg compared to the non-operated leg.

**Table 2:** Work, maximum extension and flexion torques and corresponding knee angle values (mean ± sd)

		60°/s		180°/s		Statistics*		
		operated	non-operated	operated	non-operated	leg	velocity	leg x velocity
Extension	torque [Nm/kg]	2.48 ± 0.65	2.87 ± 0.58	1.66 ± 0.40	1.88 ± 0.38	*< 0.001*	*< 0.001*	*< 0.001*
	angle [°]	62.6 ± 6.6	63.3 ± 4.6	61.5 ± 4.2	62.3 ± 3.7	0.187	0.205	0.964
	work [J/kg]	2.71 ± 0.72	3.13 ± 0.62	1.94 ± 0.49	2.17 ± 0.45	*< 0.001*	*< 0.001*	*< 0.001*
Flexion	torque [Nm/kg]	1.57 ± 0.41	1.62 ± 0.37	1.17 ± 0.33	1.23 ± 0.30	*0.004*	*< 0.001*	0.854
	angle [°]	23.6 ± 7.1	24.1 ± 5.6	37.1 ± 6.6	39.0 ± 11.5	0.180	*< 0.001*	0.462
	work [J/kg]	1.84 ± 0.51	2.01 ± 0.43	1.43 ± 0.40	1.57 ± 0.38	*0.003*	*< 0.001*	0.348
HQ-ratio		0.65 ± 0.16	0.57 ± 0.09	0.73 ± 0.22	0.66 ± 0.13	*0.001*	*< 0.001*	0.322

Note: * two factor (leg x velocity) repeated measure ANOVA, *italic - P<0.05*

**Fig. 2:**
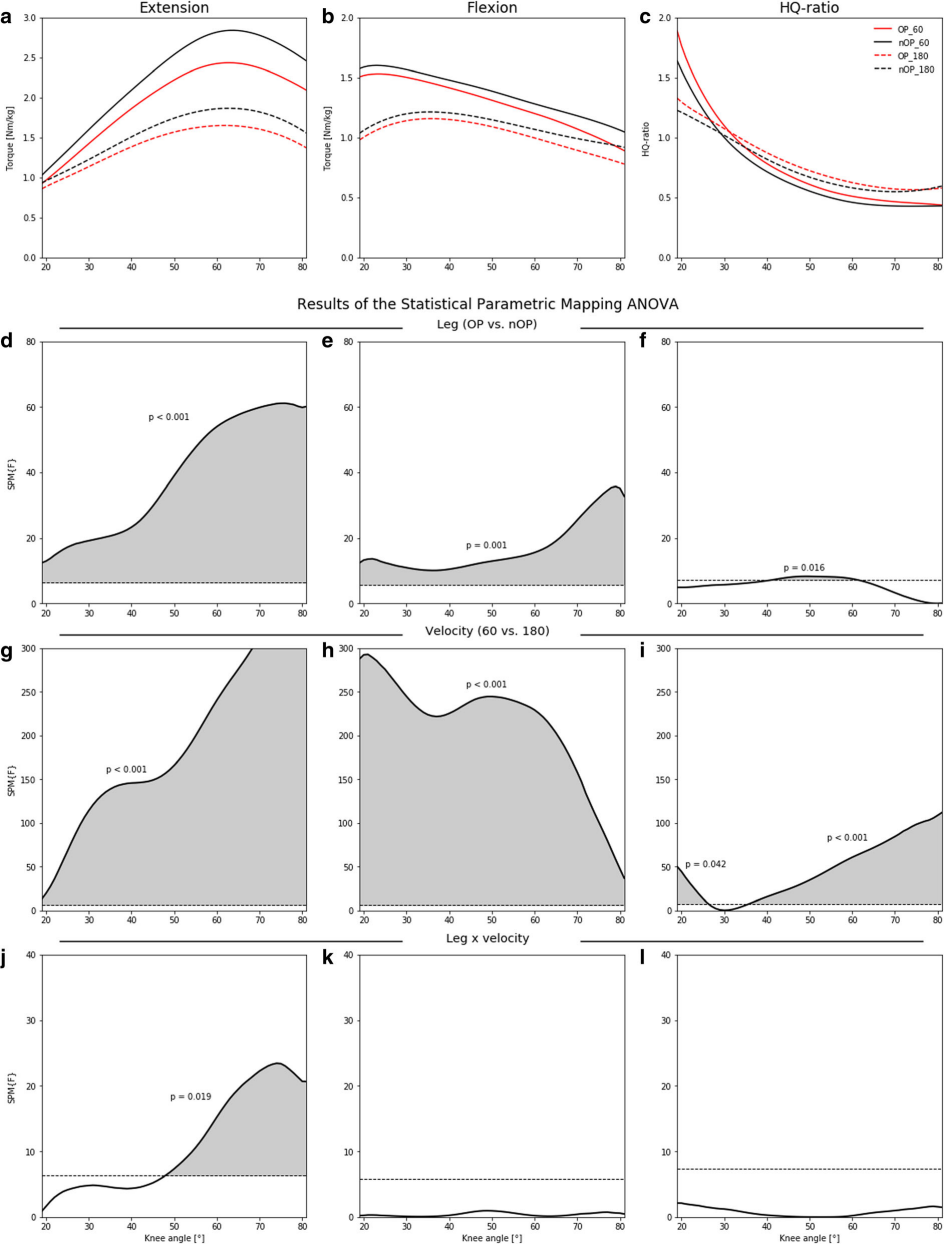
**a-c** Angle-specific extension, flexion and HQ-ratio mean curves separated for the operated (OP) and non-operated (nOP) leg as well as the 60°/s and 180°/s velocity. **d-l** Results of the two-factor (leg x velocity) repeated measure SPM ANOVA’s. Grey shaded areas marked the statistical significant regions for each factor and their interaction

The angle-specific extension, flexion and HQ-ratio mean curves and the results of the SPM ANOVA’s are shown in Fig. 2. For both extension and flexion, significant main effects for leg and velocity were found over the entire ROM (Fig. 2d, e, g, h), revealing that the torque values of the operated leg were lower than those of the non-operated as well as the values of the 60°/s were higher than those of the 180°/s. During extension, interaction effects were located in knee flexion angles greater than 50° showing that the side-to-side differences at 60°/s were greater than these at 180°/s and that the differences between the two velocities were lower in the operated leg compared to the non-operated leg (Fig. 2j). The mean angle-specific HQ-ratios ranged over the entire ROM from 0.43 to 1.89 (Fig. 2c). Main leg effects were located in knee angles between 40° to 60° (Fig. 2f). With the exception of knee angles between 27° to 35°, main velocity effects were present in the entire ROM (Fig. 2i).

The relationships between the conventional and angle-specific HQ-ratios are shown in Fig. 1 (bottom). The correlation analyses show that the resultant HQ-ratios and the angle-specific HQ-ratios were differently related depending on the knee angle and velocity. The maxima of the correlation coefficients and the corresponding knee angles for the operated and non-operated leg were 0.91 (58°) and 0.78 (60°) at the 60°/s velocity and 0.97 (53°) and 0.96 (53°) at the 180°/s velocity, respectively.

## Discussion

The aim of the study was to evaluate the usage of an angle-specific approach to analyze isokinetic torque data in patients after an ACL-reconstruction. The main outcome was that angle-specific torque as well as HQ-ratio values differ between the operated and non-operated leg and also between different movement velocities. Moreover, the angle-specific HQ-ratios over the entire ROM were not represented by the conventional HQ-ratios.

The angle-specific analysis of the mean torque-angle curves revealed significant lower values in both the operated leg and the 60°/s velocity during the extension and flexion over the entire ROM. Our results are in line with other studies, which also have also found isokinetic strength asymmetries and velocity dependent torque values in patients after an ACL-reconstruction [1, 22]. The side-to-side differences during the extension at 60°/s were larger in knee angles > 50° compared to these at 180°/s. Thus, the ability to produce high knee joint torques during the extension is impaired in the operated leg; especially, at greater knee flexion angles. In contrast, ACL-deficient patients showed the largest quadriceps deficits at knee angles < 40° for the injured leg [15, 21], where the strain of an intact ACL is significant [28]. Therefore, the reconstruction of the ACL seems to normalize the quadriceps function in lower knee flexion angles. Moreover, the altered muscle function in patients after an ACL-reconstruction may represent predominantly an adaptation to overall reduce the knee joint load and were not directly linked to the mechanical stability of the graft. Many potentially causes have been reported pertaining to the observed quadriceps deficit in the operated leg including type II muscle fiber atrophy [29], non-uniform atrophy in quadriceps muscles [30], altered knee joint mechanics [31] and/or arthrogenic muscle inhibition [32]. Future studies have to show, if a (resistance) training using individualized knee joint angles and/or specific velocities could be more effective to reduce quadriceps deficits.

In the angle-specific HQ-ratios, the SPM-ANOVA revealed a main leg and velocity effect, but not during the entire ROM. Significant differences between the operated and non-operated leg were only present in knee flexion angles from 40° to 60°, while the HQ-ratios of the operated leg were generally higher in the entire ROM. In comparison to controls, conventional calculated as well as angle-specific HQ-ratios were higher for ACL-deficient and ACL-reconstructed patients [16, 21, 33]. This deficit is more grounded on the reduced concentric quadriceps strength as on increased flexor strength of the operated leg [22]. Generally, the HQ-ratios decreased with greater knee flexion angles in both velocities, but with a different magnitude (see Fig. 2c). Around a knee angle of 30°, the HQ-ratios did not differ between the two velocities and they reached a value of nearly 1.0. This fact also shows that an angle-specific analysis can reveal more information than conventional HQ-ratios alone [21].

The angle-specific torque curves of the quadriceps and hamstring muscles have different shapes, which contributes to an angle-dependent change in H/Q-ratios [22]. Hence, conventional HQ-ratios, which were calculated using the maximum torque values, cannot represent the shape of the angle-specific HQ-ratios [16]. Moreover, the different location and velocity-dependent amount of the maximum torque values are additional arguments against the usage of conventional HQ-ratio calculations. During flexion, the velocity influences the location of the maximum torque, while it predominantly influences the amount of the maximum torque values during extension (Fig. 1 top), which has to be considered in isokinetic tests; especially, when different movement velocities are used. The linear relationships between the conventional HQ-ratios and the angle-specific HQ-ratios were generally lower in knee angles < 35° and > 65°. Moreover, the correlation coefficients were even lower at the 60°/s velocity and in the non-operated leg, respectively.

Applying SPM to isokinetic torque and HQ-ratio values in patients after an ACL-reconstruction, as shown in this study, revealed more detailed information than conventional data analyses based on single parameters [9]. Some statistical effects, which were evaluated using the conventional parameters (e.g. leg x velocity interaction effect in maximum extension torques, leg and velocity effects in HQ-ratios), were not valid for the entire ROM. Therefore, the single use of conventional data analysis has potentially led to questionable conclusions or practical applications in previous isokinetic studies. Furthermore, despite their frequently use, conventional isokinetic parameters could not be sufficiently validated as a criterion measure for a RTS decision yet [7]. Therefore, the use of angle-specific approaches is recommend for future isokinetic analyses in ACL-research. Additionally, while conventional HQ-ratios values were higher reported in patients with patella-tendon grafts and decreased during rehabilitation [7, 34], the influence of graft type and time post-surgery on the angle-specific HQ-ratios has to be investigated.

Few limitations of the study have to be acknowledged. In this study, only concentric tests were conducted. It would be of interest to use an angle-specific approach in future studies for eccentric test modes as more (functional) isokinetic indices have been developed [16, 19]. Additionally, our results cannot be extrapolated to higher velocities beyond 180°/s. However, as  only movement phases were considered, where the lever arm has reached the target velocity, the analyzable ROM would decrease significantly at higher velocities [23].

## Conclusions

In patients after an ACL-reconstruction with a hamstring tendon graft, angle-specific torques as well as HQ-ratios differ between the operated and non-operated leg, and also between different movement velocities. Moreover, the angle-specific HQ-ratios over the entire ROM were not represented by conventional calculated HQ-ratios. Therefore, the use of an angle-specific analysis of isokinetic torque values is recommended for future ACL-research, as it gives a more detailed insight into strength properties over the entire ROM, and consequently, may lead to better understanding of the muscle adaptations during the rehabilitation process. Additionally, the angle-specific approach used in the current study could be helpful in RTS decisions after an ACL reconstruction.

## Abbreviations

ACL: Anterior cruciate ligament
FDA: Functional Data Analysis
HQ: Hamstring/quadriceps
IKDC: International Knee Documentation Committee Subjective Knee Form
ROM: Range of motion
RTS: Returned-to-sport
SPM: Statistical Parametric Mapping
